# CD24 in Head and Neck Malignancies—An Uprising Biomarker?

**DOI:** 10.3390/jpm13121631

**Published:** 2023-11-22

**Authors:** Narin N. Carmel Neiderman, Shiran Shapira, Linor Klein, Dor Rafael, Gregory Gorelik, Liyona Kampel, Nadir Arber, Nidal Muhanna

**Affiliations:** 1 Head and Neck Cancer Research Laboratory, The Department of Otolaryngology Head and Neck and Maxillofacial Surgery, Tel-Aviv Sourasky Medical Center, Affiliated with the Sackler Faculty of Medicine, Tel Aviv University, Tel Aviv 6423906, Israel; carmeln@gmail.com (N.N.C.N.); linors@tlvmc.gov.il (L.K.); rafaeld@tlvmc.gov.il (D.R.); liyonakf@tlvmc.gov.il (L.K.); 2 Health Promotion Center and Integrated Cancer Prevention Center, Tel-Aviv Sourasky Medical Center, Affiliated to Sackler School of Medicine, Tel Aviv University, Tel Aviv 6423906, Israel; shiransha@tlvmc.gov.il (S.S.); nadira@tlvmc.gov.il (N.A.); 3 Pathology Department, Tel-Aviv Sourasky Medical Center, Affiliated with the Sackler Faculty of Medicine, Tel Aviv University, Tel Aviv 6423906, Israel; gorelic85@gmail.com

**Keywords:** biomarker, head and neck SCC, malignancy, early detection, CD24, PBLs

## Abstract

Introduction: CD24 is often overexpressed in human tumors as a regulator of cell migration, invasion and proliferation. It has been associated with poor prognosis and chemoresistance in laryngeal cancer. In oral cavity tumors, it was correlated with better overall survival. In this study, we aimed to evaluate the role of CD24 in peripheral blood leukocytes (PBLs) as a potential marker for head and neck malignancies. Materials and Methods: CD24/CD11b expression in peripheral blood leukocytes (PBLs) of head and neck cancer patients and matched healthy controls was analyzed via flow cytometry. Tumors and healthy tissues were immune-stained for CD24 expression and the intensity of stain was ranked. Clinical data including tumor site, size, locoregional or metastatic spread, histopathological characteristics and recurrence events were analyzed. Results: CD24 expression in PBLs was significantly higher in a cohort of 101 head and neck cancer patients compared with 101 matched healthy controls (26.9 ± 12.9 vs. 22.4 ± 13.8; *p* = 0.02). No significant differences in CD24 levels in PBLs were found between different head and neck subsites involved with malignancy. Higher CD24 levels did not correlate with any adverse feature, i.e., perineural invasion or lymphovascular invasion, advanced T stage or regional spread. Immunohistochemistry analysis demonstrated that CD24 was highly expressed in tumor tissue in comparison to healthy surrounding tissue. Conclusions: CD24 is a possible uprising marker for tumor identification, overexpressed in PBLs and is intensely stained in tumor tissue and pre-malignant lesions. Tumor–PBLs should be further studied.

## 1. Introduction

Head and neck squamous cell carcinomas (HNSCCs) are the sixth most frequently occurring cancer worldwide [1]. Although early-stage HNSCC is considered highly curable, up to 50% of patients present with advanced disease [2], associated with a high morbidity and mortality rates. Despite advances in therapy, as many as 50% of advanced HNSCC tumors relapse within the first 24 months of treatment [3]. Prognostic indicators such as histologic appearance, lymph node involvement, and the presence of distant metastasis have limited value in predicting response to a particular treatment [4].

Cluster of differentiation 24 (CD24) is a small heavily glycosylated glycosylphosphatidylinositol-linked cell surface protein, a ligand for P-selectin, which is broadly expressed on B-cells and neuroblasts. While the first description of its use in the context of malignancy was made as early as 1987 [5], its applications in tumor biology and tumor prognosis has gained interest only in recent years. It is expressed not only in hematological malignancies, but in a wide array of solid tumors [6]. In recent years, CD24 gene has raised interest predicting tumor behavior and treatment response. CD24 expression causes the acquisition of cellular properties associated with tumor proliferation, growth and metastasis [7], and its potential role in malignancy is yet to be determined. These associations were further established in head and neck cancer cell lines: CD24+ cells possessed stemness characteristics of self-renewal and differentiation, a higher cell invasion in vitro and made a higher number of colonies in collagen gels compared to CD24− HNSCC cells. In addition, the CD24+ cells were more chemo-resistant to gemcitabine and cisplatin compared to CD24− cells [8]. Furthermore, CD24 was recently shown to promote immune escape among cancerous cells. CD24 expression on the surface of tumor cells interacts with Siglec-10, which is expressed on immune cells, promoting tumor immune escape through corresponding molecular mechanisms in vitro among T and B lymphocytes, macrophages and NK cells [9].

In the head and neck cancer field, the expression of CD24 was found to be associated with unfavorable outcomes in laryngeal cancer patients [10]. High CD24 expression was significantly associated with worse clinical T stage, lymph node metastasis and tumor size. Patients with laryngeal squamous cell carcinoma recurrence had higher levels of CD24 protein than those who did not experience a recurrence. Univariate and multivariate analyses revealed that CD24 was a significant predictor for decreased overall survival [10]. Modur V. et al. further showed that CD24 expression level appears to be a significant molecular phenotype of cisplatin-resistant residual cells in laryngeal carcinoma lines. CD24 expression level directly affects cisplatin sensitivity and affects the expression of apoptotic, stem and drug resistance genes. A relatively small retrospective patient tumor analysis suggests that tumors with a high expression of CD24 show an unfavorable response to cisplatin treatment [11]. These findings further correspond with similar in vitro findings by the same research group, demonstrating that higher expression of CD24 in head and neck tumors result in a cisplatin resistant population that may well be the cause of unfavorable response to cisplatin treatment [12].

The peripheral blood cells are the body’s sensors: traveling through the body, entering and exiting tissues and detecting changes in them. As a result of these changes and cell-to-cell interactions, the expression of the various biomarkers changes within the various immune system cells, reflecting and possibly alerting of cancerous cells’ presence [13]. In 2015, Kraus S. et al. demonstrated that CD24 levels in peripheral blood leukocytes (PBLs) may be a potential marker of malignancy. Protein extracted from PBLs was subjected to immunoblotting using anti-CD24 monoclonal antibodies, showing that the sensitivity and specificity of CD24 for distinguishing colorectal cancer from normal subjects were 70.5% (95% CI, 54.8–83.2%) and 83.8% (95% CI, 74.6–92.7%), respectively. Further evidence supporting the possible role of CD24 in PBLs as a biomarker for malignancy are still under investigation with promising results [14].

The main aim of our study was to evaluate the potential role of CD24 levels in PBLs as a biomarker for malignancy. We further aimed to seek if head and neck cancer patients with high CD24 levels had certain adverse features or poorer outcomes.

## 2. Materials and Methods

### 2.1. Subjects

Blood samples were obtained between 2015 and 2022 from consecutive healthy volunteers, of ages 20–85, presenting for a comprehensive physical examination at the Health Promotion and Integrated Cancer Prevention Center (HP-ICPC), and perioperatively from head and neck cancer patients from the department of head and neck surgery and maxillofacial and otolaryngology at Tel Aviv Sourasky Medical Center, Tel-Aviv, Israel. Subjects unable or unwilling to provide informed consent, those who had co-existing signs or symptoms of active inflammation, autoimmune background, hematologic malignancies in the present or past, or melanotic lesions, immunosuppression due to medical treatment or under the influence of chemotherapy or immunotherapy, were excluded from the study. Eligible subjects completed a detailed questionnaire on medical history, including cancer diagnoses and demographic data. Data regarding tumor TNM and final staging, recurrence events, prior chemoradiation treatment, tumor size and depth of invasion (DOI) in subjects with oral cavity, as well as adverse pathological features (lymphovascular invasion, perineural invasion, extra nodal extension) were recorded. Tumor characteristics (T), nodal spread (N) and distant metastasis (M) (TNM) [15] staging were determined by a medical oncologist and a head and neck surgeon after multidisciplinary tumor board discussion, according to the American Joint Committee of Cancer (AJCC) guidelines [16]. Blood specimen collection and handling were performed using a standard hospital procedure and a set of protocols. Patient information was deidentified and only anonymized data were available to the investigators.

Written informed consent was obtained from all eligible participants prior to inclusion in the study. Approval for this study was provided by the Institutional Review Board (IRB TLV 02-130) of Tel Aviv Sourasky Medical Center, Tel Aviv, Israel and the Israeli Ministry of Health.

### 2.2. Isolation of Peripheral Blood Leukocytes

Blood was collected into standard 9 mL collection tubes (Vacuette^®^, Greiner bio-one, Kremsmünster, Austria). All samples were collected and processed in an identical manner. PBLs were isolated from whole blood samples by collecting buffy coats obtained after blood centrifugation for 3 min at 3000 rpm and discarding the plasma supernatant. Residual erythrocytes were lysed via brief incubation in erythrocyte lysis buffer containing 155 mM NH_4_Cl, 0.1 mM EDTA and 10 mM KHCO_3_ (30–40 min on ice) and then via centrifugation at 3000 rpm, 4 °C for 5 min. Clean leukocytes pallets were obtained after one or two more washes with ELB. PBLs underwent fixation with formaldehyde solution (2% FA in PBS), 15 min at RT, and stored at 4 °C until use.

### 2.3. Flow Cytometry

Leukocytes of 1 × 10^6^ were used for each test and were stained with 0.05 µg of anti-CD24−FITC mAb (NS17-FITC), anti-CD11b−PerCp-Cy5.5 (purchased from Abcam), diluted in FACS buffer or remained unstained for 30 min at a room temperature. Then, the cells were washed twice with FACS buffer (0.01% sodium azide, 10% fetal bovine serum (FBS) in ice-cold PBS) and then analyzed via a flow cytometry device (CyFlow Cube 6, Sysmex, Germany). Data were analyzed following the creation of a hierarchical population tree. This template was used in all subsequent analyses. The template file includes compensation adjustment, which is uniformly applied to all the data collected in order to minimize fluorescence overlap between detection channels. The percentage of CD24-positive cells (CD24+) was calculated by subtracting CD24+/CD11b+ cells (dual stain) from CD24+ cells (single stain). Following extensive screening and testing of various biomarkers and their combinations, we found a sub-population of biomarkers that successfully discriminates between healthy subjects and cancer patients with high sensitivity and specificity.

### 2.4. FITC Conjugation

Purified anti-CD24 humanized mAb was developed by our laboratory personnel and was reliably used in previous studies. High-affinity antibodies were selected and created from combinatorial phage-displayed antibody libraries that contain varying degrees of diversity at randomized positions. A chosen matured clone was isolated and showed a higher binding strength, compared to the parental murine and humanized Abs. The matured antibody showed selective recognition and binding to the CD24 antigen, which proves that the genetic manipulations carried out did not affect its properties.

Anti-CD24 humanized mAb was labeled with FITC (ThermoFisher scientific, Waltham, MA, USA) according to manufacturer instructions. Briefly, the antibody was diluted in PBS to a final concentration of 2 mg/mL (in 0.5 mL) and 40 µL of the Borate buffer (0.67 M) was added. The FITC reagent was then added to the prepared mAb and mixed until all the dye was dissolved. The vial was briefly centrifuged and the reaction mixture was incubated for 60 min at room temperature protected from light. Then, the labeling reaction was loaded on purification resin and the labeled mAb was isolated via centrifugation and kept at −20 °C.

### 2.5. Immunohistochemistry

Patients’ formalin-fixed paraffin-embedded slides were retrieved and reviewed by a head and neck pathologist to select blocks that contained the tumor. Formalin-fixed paraffin-embedded slides were freshly cut (4 Micron) from the selected blocks. Sections were mounted on superfrost slides (Menzel Gläser, Braunschweig, Germany), deparaffinized with xylene and rehydrated gradually. Antigen retrieval was achieved via pressure cooking in 0.01 mol/L citrate buffer for 5 min. The primary CD24 antibody (SWA11 was diluted 1:100 using a background reducing dilution buffer (DAKO, Hamburg, Germany) and incubated with HRP-conjugated Goat Anti-Mouse IgG(H + L) at room temperature for 1 h. Afterward, the slides were briefly counterstained with hematoxylin and aqueously mounted.

Immunostaining of tissue slides was independently evaluated by an experienced head and neck clinical pathologist who was unaware of patient tumor status, subsite and outcome. The pathologist ranked the intensity of tumor stain and background healthy tissue from 0 (no stain) to 10 (intense stain).

### 2.6. Matching and Statistical Analysis

Cancer patients were matched with healthy subjects who underwent screening at the HP-ICPC according to age and gender. Patients with an unclear background, benign tumors or inflammation were excluded from the ‘Healthy’ group. The preferred method of analysis was parametric using *t*-test, when parametric analysis was not applicable, the non-parametric Mann–Whiteny test was used for variable comparison. Categorical variables were compared using the chi-squared test or Fisher exact test when needed.

Currently, an established cut-off value for CD24 expression levels in peripheral blood cells is yet to be established. CD24+/CD11b− PBLs’ high percentage was arbitrarily defended at the 10th percentile of our cohort. Given these data, we have examined the distribution of this value in our cohort and have defined the high CD24 expression level as exceeding values of 36.86%, which represented the upper 10th of the cohort.

Sample size: Our study included 101 cancer patients matched by age and gender to 101 healthy participants from the ICPC database. Given the normal distribution of CD24 levels in the general population, our matched samples size would suffice to reject the null hypothesis with a probability (power) of 84.7% for an intergroup delta as small as 0.6% with standard deviation of 2%. The type I error probability associated with this samples size (alpha) is 5%.

All statistical tests were performed at α = 0.05 (two-sided). All *p*-values reported were rounded to two decimal places. Data were analyzed using IBM SPSS Statistics software (IBM SPSS statistics for Windows, version 28; IBM Corp., New York, NY, USA, 2021).

## 3. Results

Our study included 101 patients with different head and neck malignancies and 101 matched controls presented at the Health Promotion and Integrated Cancer Prevention Center database (Table 1). Mean age was 63.71 ± 12.5 years, with male predominance (n = 144, 71.3%). Flow cytometry analysis revealed a higher percentage of PBLs expressing CD24+/CD11b− among patients with head and neck malignancies compared with healthy controls (26.9 ± 12.9 vs. 22.4 ± 13.8% of positive percentage of CD24^+^/CD11b^−^ cells; *p* = 0.02).

**Figure 1:**
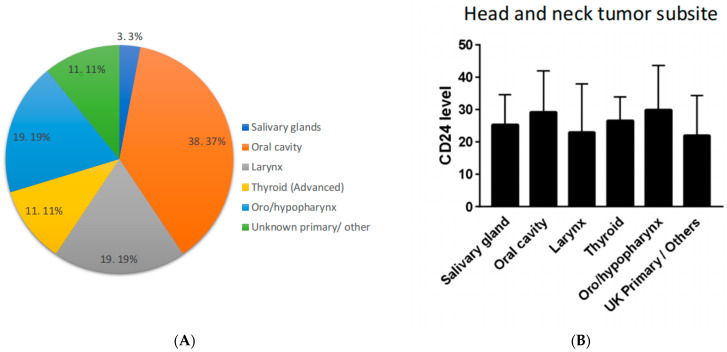
Cohort tumor characteristics according to tumor subsites in the head and neck system. (**A**) A pie chart of the different subsites obtained, presented as N, (%). (**B**) CD24+/CD11b− average percentage ± standard deviation obtained at the different subsites in the head and neck system, *p* = non-significant.

Different subsites of head and neck involvement are specified in Figure 1A with the majority of the tumors being SCCs of the head and neck. No significant differences in the percentage of PBLs expressing CD24+/CD11b− in flow cytometry were found between the different head and neck sites involved with malignancy (Figure 1B).

We further intended to decipher unique characteristics of HN malignancy patients who expressed higher percentage CD24+/CD11b− among PBLs in flow cytometry. Male gender was found to be more frequent among patients with higher percentage CD24^+^/CD11b− PBLs and patients with HN malignancy (n = 54 (66.7%) vs. n = 18 (90%), *p =* 0.04). Interestingly, neither a higher T stage nor N stage correlated with an increased percentage of CD24+/CD11b− expression in PBLs’ flow cytometry (Table 2 and Table 3). Locoregional disease was not found to increase the percentage of CD24+/CD11b− expression in PBLs in flow cytometry. Other histopathological characteristics of the tumor, such as perineural invasion, did not affect the percentage of PBLs expressing CD24+/CD11b− in flow cytometry. These results were consistent in the subgroups of HNSCC (Table 4). As for clinical characteristics implying an aggressive disease, i.e., future recurrence, no statistical difference was found (Table 4).

**Figure 2:**
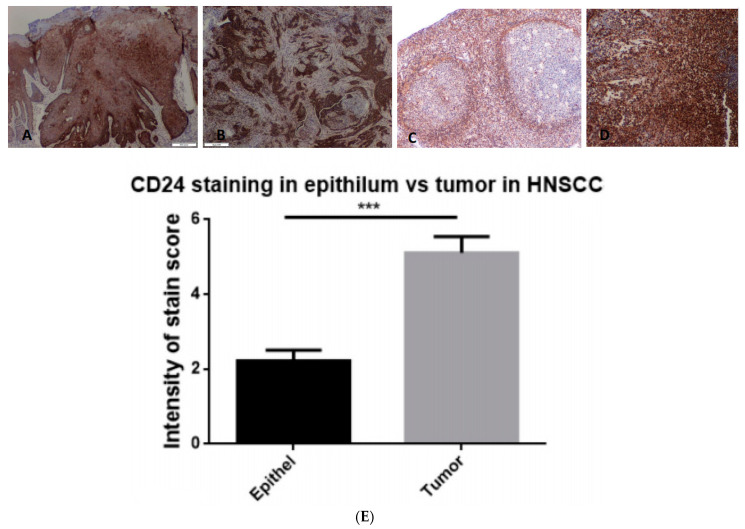
Immunohistochemical staining of head and neck malignant tumor tissue for CD24. (**A**) Oral cavity squamous cell carcinoma stained for CD24; (**B**) hypopharyngeal squamous cell carcinoma stained for CD24; (**C**) laryngeal squamous cell carcinoma stained for CD24; (**D**) salivary gland mucoepidermoid carcinoma; (**E**) stain intensity score grading for tumor vs. healthy surrounding tissue. *** *p* value < 0.001.

**Figure 3:**
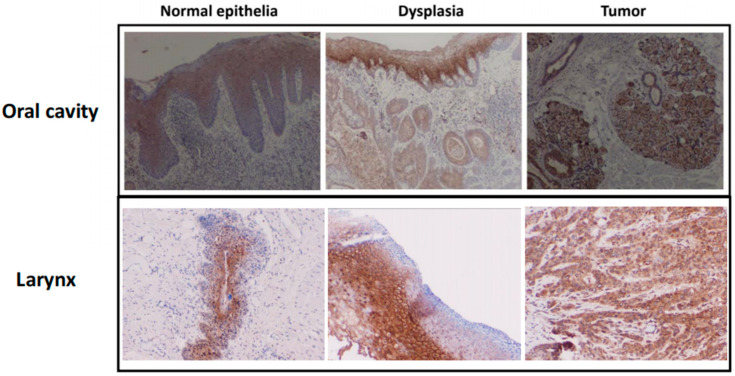
Intensity of CD24 in immunohistochemistry staining increases with degree of dysplasia in head and neck squamous cell carcinoma.

We further aimed to evaluate the presence of CD24 protein in the tumor tissue of HNSCC patients of our cohort, in comparison to healthy margin tissues. Stain scoring by a blinded pathologist demonstrated that HNSCCs were characterized with higher scores of CD24 stain intensity in comparison to healthy tissue (*p* < 0.05, Figure 2). Interestingly, throughout the pathological assessment, we observed positive stain not only in the tumor, but also in dysplastic lesions. In clinical histopathological observation, it was found to be less intense compared to tumor tissue, but higher than the healthy tissue (Figure 3).

## 4. Discussion

In our cohort, flow cytometry analysis revealed a higher number of leukocytes expressing a significantly higher percentage of CD24+/CD11b− PBLs among HN cancer patients peripheral blood samples and in immunohistochemistry of tumor tissue compared with normal controls. However, we did not detect any unique characteristic of patients with a high percentage of CD24+/CD11b− among PBLs, or correlation to tumor characteristics, apart from male gender.

The role of CD24 in head and neck malignancy is yet to be determined. In vivo mice models of oral cavity showed that, in mice with dorsal skinfold chamber sponges seeded with CD24^+^ cells, there is a significantly higher functional capillary density in comparison to those seeded with CD24^−^ cells [17]. High functional capillary density was found in 1971 as a marker for tumor growth and the development of solid tumors, indicating accelerated angiogenesis and tumor progression [18]. Interestingly, in oral squamous cell carcinoma, CD24 expression was also not associated with tumor size, histological differentiation, or lymph node metastasis. However, there was an association between CD24 expression and invasion mode. Authors suspected the decreased E-cadherin expression was induced via CD24 overexpression [19]. These results stand in line with our study that did not find a correlation to tumor or patient properties.

In contradiction to our results, Fugle et al. showed, in 2016, a protective effect of CD24 in oral cavity malignancy. High CD24 levels were found to blunt oral squamous cancer development and dampen the functional expansion of myeloid-derived suppressor cells. CD24 deficiency increased the oral cavity tumor burden in response to the carcinogen 4-nitroquioline 1-oxide. Immune profile analysis showed a significant expansion as well as an increased suppressive function of myeloid-derived suppressor cells (MDSCs) in CD24−/− mice. Orthotopically transplanted syngeneic squamous carcinoma model in the tongue of CD24−/− and CD24+/− mice confirmed the protective roles of CD24 against cancer. Moreover, the difference in tumor growth between CD24−/− and CD24+/− mice was blunted by immunodepleting of MDSCs [20]. These results are further supported by Ghuwalewala et al., who also addressed CD44 expression in oral tongue population, showing that CD24 showed a significantly lower expression in tumor tissues. The authors concluded that CD44 (high)/CD24 (low) represents cancer stem-like cells in oral squamous cell carcinoma [21]. These results do not stand in line with the high intensity of immunohistochemistry staining seen in oral cavity SCC in our cohort.

These results are in inverse relation to the ones found by Modur et al., showing that CD24 presence may lead to unfavorable cisplatin treatment outcome in laryngeal carcinoma cell lines, with a direct effect on cisplatin sensitivity and an indirect effect on apoptotic stem and drug resistance genes. It was found to be a possible indicator to the response to platinum-based therapies [11]. In our study, we did not detect any correlation between SCC recurrence or treatment failures and high CD24 levels.

Interestingly, CD24 is uncorrelated to patient characteristics, not only among cancer patients, but also among healthy controls. Two decades ago, initial reports showed that in the early stages of the multi-step carcinogenesis process, CD24 rises. Since then, many groups and many papers support the use of CD24 as a marker for prognosis and predicting clinical outcome in many tumor types. Its behavior from early stages to late stages of malignancy was broadly investigated by Kraus et al. As a biomarker for the early detection of cancer, CD24 is over expressed at the early stages of the disease, for example, at the polyp stage in colorectal cancer. There are therefore no significant differences in its levels throughout the different stages of the disease [22]. We have tested the association between CD24/CD11b expression and clinical measurements, healthy behaviors (smoking, physical activity) and demographic characteristics among healthy participants. These results demonstrated that the expression of CD24 is not dependent on a subject’s clinical measurements, behaviors (smoking, physical activity) and demographic characteristics [14]. 

## 5. Study Limitations

Our study has several limitations. First, we had a limited cohort of patients gathered in a single center, with a wide heterogeneity of tumor sites. However, we did not detect any correlation between CD24 levels and tumor location, in contradiction to the findings of Modur et al. [12] and Fugle et al. [20] that noted contradictory trends regarding oncologic outcome and overexpression of CD24 in different subsites of HNSCC.

Moreover, CD24 was reported to express in various other malignant conditions, including B-cell lymphomas, gliomas, lung, breast, urothelial tumors and pancreatic cancer [23,24] and a second primary affecting the CD24 result cannot be excluded. It should be noted that as part of the oncologic workup, all of our patients underwent PET-CT prior to surgical intervention to detect distant metastasis, that would probably reveal a second primary. However, PETCT resolution is still limited; therefore, very early stage or microscopic malignancy under PETCT resolution may be missed.

Third, bias can be introduced from a number of sources, including the study population itself, the metabolic state of the subject prior to blood collection and during the processing of serum, storage and analysis [6]. However, the role of CD24 as a possible biomarker was previously discussed and widely established by Shapira and Arber et al. [14,22] and their results were replicated widely in multiple continuous studies. Fourth, we did not address tumor microenvironment-and-leukocytes interaction, which may relate to CD24 presence, and did not evaluate HPV status.

In conclusion, CD24 was found to be increased in HN malignancy, and may have a possible pathophysiological role in the evolution of HN malignancy. HN malignancies are usually diagnosed at an advanced stage, with poor prognosis and outcomes. Advanced-stage presentation is a major limitation to surgical treatment, stressing the necessity of a noninvasive marker for early detection. In our study, we detected high CD24 levels not only in the tumor, but also in PBLs of patients with HN tumors, similarly to the work of Kraus S et al. [22]. CD24 alone, or in a panel of serologic markers, may be a very useful clinical tool in screening asymptomatic individuals for head and neck malignancy and would better guide in the utilization of resources for endoscopic and examination under anesthesia evaluation. Tumor–PBL interaction has promising potential for early detection among patients with pre-malignant lesions and should be further studied.

## Figures and Tables

**Table 1:** Cohort characteristics and CD24+/CD11b− PBLs percentage in healthy and head and neck malignancy patients (N = 202).

Variable	Entire Cohort (n = 202)	Patients (n = 101)	Healthy Controls (n = 101)	*p*-Value
Age, mean (SD), y	63.71 (12.5)	63.82 (12.49)	63.6 (12.57)	0.9
Gender, male, No. (%)	144 (71.3)	72 (71.3)	72 (71.3)	1
CD24, mean (SD), % of positive PBLs expressing CD24+/CD11b−	24.68 (13.57)	26.97 (12.92)	22.39 (13.89)	0.02
CD24 mean (SD), no. of patients with 10th top percentile of positive PBLs expressing CD24+/CD11b−	34 (16.8%)	20 (19.8)	14 (13.9)	0.26

SD = Standard deviation; No. (%) = Number (%).

**Table 2:** HN malignancy patients with high vs. low CD24+/CD11b− PBLs percentage.

Variable		Entire Cohort (n = 101)	CD24+/CD11b− PBLs Non High Percentage (n = 81)	CD24+/CD11b− PBLs High Percentage (n = 20)	*p*-Value
Age, mean (SD), y		63.82 (12.49)	64.09 (13.15)	62.75 (9.56)	0.56
Gender, male, n (%)		72 (71.3)	54 (66.7)	18 (90)	0.04
CD24, mean (SD), % of positive cells		26.97 (12.92)	22.42 (9.9)	45.39 (4.17)	n/a
Smoking status, n (%)	Never	37 (36.6)	33 (40.7)	4 (20)	0.19
	Active	36 (35.6)	28 (34.6)	8 (40)	
	Past	28 (27.7)	20 (24.7)	8 (40)	
Tumor type, n (%)	Salivary glands	3 (3)	3 (3.7)	0 (0)	n/a
	Oral cavity	38 (37.6)	28 (34.6)	10 (50)	
	Larynx	19 (18.8)	16 (19.8)	3 (15)	
	Thyroid	11 (10.9)	10 (12.3)	1 (5)	
	Oro/hypopharynx	19 (18.8)	14 (17.3)	5 (25)	
	Unknown primary/other	11 (10.9)	10 (12.3)	1 (5)	
Histopathological classification, n (%)	SCC	83 (82.2)	65 (80.2)	18 (90)	n/a
	MEC	4 (4)	4 (4.9)	0 (0)	
	Sarcoma	1 (1)	0 (0)	1 (5)	
	PTC	11 (10.9)	10 (12.3)	1 (5)	
	Other	2 (2)	2 (2.5)	0 (0)	
T classification, n (%)	0–2	50 (50.5)	40 (50.6)	10 (50)	<0.99
	3–4	49 (49.5)	39 (49.4)	10 (50)	
N classification, n (%)	0	52 (53.1)	42 (53.2)	10 (52.6)	0.97
	1–3	46 (46.9)	37 (46.8)	9 (47.4)	
Tumor size, mean (SD), mm		24.06 (12.92)	22.9 (16.95)	30.11 (27.94)	0.53
Depth of invasion, mean (SD), mm		7.67 (6.35)	7.72 (6.47)	7.49 (6.27)	0.93
Extra nodal extension, n (%)		9 (25)	7 (23.3)	2 (33.3)	0.63
Total metastasis, median (IQR)		1 (0–1)	1 (0–1)	0 (0–1)	0.47
Total lymph nodes dissected, median (IQR)		21 (12–35)	19.5 (9.5–31.75)	24 (23–41)	0.04
Angiolymphatic invasion, n (%)		2 (3.5)	2 (4.3)	0 (0)	<0.99
Perineural invasion, n (%)		12 (19)	10 (19.6)	2 (16.7)	<0.99
Recurrence, n (%)		28 (28.9%)	23 (29.9%)	5 (25%)	0.79
Adjuvant treatment, n (%)		56 (56%)	46 (57.5%)	10 (50%)	0.55

SD = Standard deviation; MEC = mucoepidermoid carcinoma; SCC = squamous cell carcinoma; PTC = papillary thyroid cancer; n/a = not available; T = tumor, classified as Tis to T4 (TNM clinical classification, AJCC 8th edition); N = nodes, classified as N0 to N3 (TNM clinical classification, AJCC 8th edition).

**Table 3:** HNSCC cohort characteristics of patients with high vs. low CD24+/CD11b− PBLs percentage.

Variable		Entire Cohort (n = 83)	CD24+/CD11b− PBLs Non High Percentage (n = 65)	CD24+/CD11b− PBLs High Percentage (n = 18)	*p*-Value
Age, mean (SD), y		64.66 (11.52)	65.09 (12.29)	63.11 (8.3)	0.52
Gender, male, n (%)		66 (79.5)	49 (75.4)	17 (94.4)	0.1
CD24, mean (SD), % of positive cells		27.19 (13.46)	22.14 (10.37)	45.44 (4.3)	n/a
Smoking status, n (%)	Never	27 (32.5)	24 (36.9)	3 (16.7)	0.16
	Active	32 (38.6)	25 (38.5)	7 (38.9)	
	Past	24 (28.9)	16 (24.6)	8 (44.4)	
Tumor type, n (%)	Oral cavity	38 (45.8)	28 (43.1)	10 (55.6)	n/a
	Larynx	17 (20.5)	14 (21.5)	3 (16.7)	
	Oro/hypopharynx	19 (22.9)	14 (21.5)	5 (27.8)	
	Unknown primary/other	9 (10.8)	9 (13.8)	0 (0)	
T classification, n (%)	0–2	38 (45.8)	29 (44.6)	9 (50)	0.69
	3–4	45 (54.2)	36 (55.4)	9 (50)	
N classification, n (%)	0	46 (56.1)	37 (56.9)	9 (52.9)	0.77
	1–3	36 (43.9)	28 (43.1)	8 (47.1)	
Tumor size, mean (SD), mm		23.56 (19.58)	22.42 (17.04)	28.88 (29.61)	0.85
Depth of invasion, mean (SD), mm		7.67 (6.35)	7.72 (6.47)	7.49 (6.27)	0.93
Extra nodal extension, n (%)		7 (28)	6 (30)	1 (20)	<0.99
Total metastasis, median (IQR)		0 (0–1)	0.5 (0–1)	0 (0–1)	0.44
Total lymph nodes dissected, median (IQR)		22 (12–36)	19 (11.5–35.5)	29.5 (23–41.75)	0.08
Angiolymphatic invasion, n (%)		0 (0)	0 (0)	0 (0)	n/a
Perineural invasion, n (%)		10 (20.4)	8 (20.5)	2 (20)	<0.99
Recurrence, n (%)		24 (30)	30 (32.3)	4 (22.2)	0.41
Adjuvant treatment, n (%)		46 (55.4)	38 (58.5)	8 (44.4)	0.29

HNSCC = Head and neck squamous cell carcinoma; SD = standard deviation; n/a = not applicable; T = tumor, classified as Tis to T4 (TNM clinical classification, AJCC 8th edition); N = nodes, classified as N0 to N3 (TNM clinical classification, AJCC 8th edition).

**Table 4:** Pearson correlation between patient and tumor characteristics and CD24+/CD11b− PBLs percentage.

	Correlation with CD24+/CD11b− Percentage in PBL	*p*-Value
Age (years)	−0.099	0.162
Gender	0.116	0.100
Smoking status	0.117	0.242
Recurrence of tumor	−0.036	0.719
Tumor size (mm)	0.006	0.967
Depth of invasion (mm)	−0.106	0.522
Angiolymphatic invasion	0.039	0.771
T classification	−0.010	0.920
N classification	0.042	0.680

mm = Millimeter; T = Tumor, classified as Tis to T4 (TNM clinical classification, AJCC 8th edition); N = Nodes, classified as N0 to N3 (TNM clinical classification, AJCC 8th edition).

## Data Availability

Data supporting reported results will be sent upon request from Dr. Narin N. Carmel Neiderman.
